# A membrane-targeting magnolol derivative for the treatment of methicillin-resistant *Staphylococcus aureus* infections

**DOI:** 10.3389/fmicb.2024.1385585

**Published:** 2024-05-17

**Authors:** Fushan Zhang, Hui Fang, Yuxin Zhao, Buhui Zhao, Shangshang Qin, Yu Wang, Yong Guo, Jifeng Liu, Ting Xu

**Affiliations:** ^1^Department of Clinical Laboratory, The First Affiliated Hospital of Zhengzhou University, Zhengzhou, China; ^2^School of Pharmaceutical Sciences, Zhengzhou University, Zhengzhou, China; ^3^Hunan Province Cooperative Innovation Center for Molecular Target New Drug Study, School of Pharmaceutical Science, Hengyang Medical School, University of South China, Hengyang, China

**Keywords:** magnolol, methicillin-resistant *Staphylococcus aureus*, antimicrobial activity, antibacterial, bacterial cell membrane

## Abstract

Multidrug-resistant bacterial infections are a major global health challenge, especially the emergence and rapid spread of methicillin-resistant *Staphylococcus aureus* (MRSA) urgently require alternative treatment options. Our study has identified that a magnolol derivative **6i** as a promising agent with significant antibacterial activity against *S. aureus* and clinical MRSA isolates (MIC = 2–8 μg/mL), showing high membrane selectivity. Unlike traditional antibiotics, **6i** demonstrated rapid bactericidal efficiency and a lower propensity for inducing bacterial resistance. Compound **6i** also could inhibit biofilm formation and eradicate bacteria within biofilms. Mechanistic studies further revealed that **6i** could target bacterial cell membranes, disrupting the integrity of the cell membrane and leading to increased DNA leakage, resulting in potent antibacterial effects. Meanwhile, **6i** also showed good plasma stability and excellent biosafety. Notably, **6i** displayed good *in vivo* antibacterial activity in a mouse skin abscess model of MRSA-16 infection, which was comparable to the positive control vancomycin. These findings indicated that the magnolol derivative **6i** possessed the potential to be a novel anti-MRSA infection agent.

## 1 Introduction

The global health crisis is significantly intensified by multi-drug resistant bacteria (MDR), especially during the COVID-19 pandemic (Magana et al., 2020; Nourollahpour Shiadeh et al., 2022). By 2050, MDR is expected to cause 10 million deaths per year and an astounding US $100 trillion in economic losses (Martin et al., 2019; Sulis et al., 2022). *Staphylococcus aureus* is a major pathogen notorious for causing severe infections such as pneumonia and sepsis, and its resistance to most antibiotics makes it particularly dangerous. Infections with strains such as methicillin-or vancomycin-resistant *Staphylococcus aureus* contribute to high mortality rates worldwide (Ayukekbong et al., 2017; Martin et al., 2019; Sulis et al., 2022; Lin et al., 2023). With limited antibiotics effective against these resistant strains, there’s a pressing need for new drugs with unique mechanisms to tackle this challenge.

Antimicrobial peptides (AMPs), as part of host defense systems in a variety of animals, plants, and bacteria, exhibit unique antimicrobial mechanisms that can reduce the possibility of bacterial resistance (Lazzaro et al., 2020; Alghamdi et al., 2021). Unlike conventional antibiotics, AMPs exhibit high specificity by targeting specific bacterial elements such as phosphatidylglycerol (PG), which accumulate on the bacterial surface through electrostatic interactions and disrupt membrane stability and integrity (Zhang and Gallo, 2016). In addition, natural products represent a valuable library of compounds, and many potent and unique mechanisms of small molecule antimicrobial drugs are derived from endogenous antimicrobial metabolites of plants (Yoshikawa et al., 2012; Qian et al., 2015; Tagare and Vaidyanathan, 2018; Tóth et al., 2018; Yan et al., 2018; Zhang et al., 2022). Our group previously created a series of AMP mimetics using magnolol, a bioactive compound from *Magnolia officinalis* identified to have initial antimicrobial activity, as the core structure through a modular assembly strategy, overcoming its poor water solubility and high biotoxicity (Guo et al., 2021a). However, the specific antimicrobial mechanism of the notably active component **6i** remains to be elucidated.

In this study, we tested the antimicrobial effects of **6i** by a series of *in vitro* assays, including MIC and MBC assays, assessment of hemolytic activity, and membrane selectivity studies. In addition, the kinetics of killing bacteria and the potential for resistance development were investigated, which is crucial because the ability to circumvent resistance mechanisms is a key consideration in the development of new antibiotics. In addition, we investigated its ability to disrupt MRSA biofilms. Given the role of biofilms in chronic infections and antibiotic resistance, understanding this ability is essential for the potential application of compounds. Scanning electron microscopy and fluorescence microscopy were used to further explore the antibacterial mechanism of action, providing insights into the interaction with bacterial cells at the molecular level. Finally, after verifying its stability in plasma, its safety and efficacy *in vivo* were evaluated, ensuring its suitability for further development as a therapeutic agent. By elucidating the potential of compound 6i against MRSA, it provides a promising avenue against MDR.

## 2 Materials and methods

### 2.1 General information

All of the reagents and other solvents were purchased commercially and used without further purification. The ^1^ H/^13^C NMR spectra of derivative **6i** were determined using a Bruker Avance 400 or 600 MHz instrument. The purity of **6i** was determined by HPLC. An Agilent 6545 electrospray ionization quadrupole time-of-flight mass spectrometer (ESI-Q-TOF LC/MS) was used to identify high-resolution mass spectra (HRMS) of the **6i**.

### 2.2 Synthesis of compound **3**

To a solution of the n-Butylamine (**1**, 10.0 mmol) and K_2_CO_3_ (15.0 mmol) in CH_2_Cl_2_ at 0°C under N_2_, bromoacetyl bromide (15.0 mmol, 3.0 g) was added dropwise. After stirring for approximately 0.5 h, the reaction was removed to room temperature and stirred for 3–5 h. When the reaction was complete according to thin-layer chromatography (TLC) detection, the reaction mixture was filtered and distilled to give **2**, which was directly used for the next step. Subsequently, to a solution of **2** and K_2_CO_3_ (15.0 mmol) in acetone, an aqueous solution of dimethylamine (15.0 mmol) was added. After stirring at 25°C for 12–18 h, the reaction mixture was filtered and concentrated. The concentrate was isolated by silica-gel column chromatography (CC) to obtain compound **3**.

### 2.3 Synthesis of compound **5**

Magnolol (**4**, 0.2 mmol) and the bromoacetyl bromide (0.6 mmol) were dissolved in dry acetone in a round flask, K_2_CO_3_ (0.6 mmol, 82.9 mg) was added, and the mixture was stirred at 57°C. The mixture was filtered to remove K_2_CO_3_ and concentrated when stirred for approximately 24 h. Finally, preparative thin-layer chromatography (PTLC) was used to purify the concentrate and obtain intermediate **5** in yield of 53%.

### 2.4 Synthesis of compound **6i**

Compound **5** (0.2 mmol) and compound **3** (0.6 mmol) were dissolved in MeOH, and then the mixture was stirred at reflux. After the reaction was completed, the mixture was evaporated under reduced pressure, and the residue was purified by PTLC or flash chromatography [eluting solvents: CH_2_Cl_2_/MeOH (v/v) = 25:1–10:1] to afford the title compound **6i**.

### 2.5 Determination of MICs and MBCs

The Minimum Inhibitory Concentration (MIC) and Minimum Bactericidal Concentration (MBC) of compound **6i** were determined by the micro broth dilution method with reference to the CLSI standard. The Single colonies of *Staphylococcus aureus* ATCC 29213 and 10 clinically isolated strains MRSA were selected and placed in 1 mL MHB medium, respectively, and cultured at 37°C and 200 rpm for 3 h, and then diluted to a concentration of 1.0 × 10^5^ CFU/mL. Compound **6i** (0.5–256 μg/mL) and positive control vancomycin (0.0625–32 μg/mL) diluted with MHB medium were added to 96-well plates respectively, and then diluted bacterial solution was added successively. After incubation at 37°C for 16–20 h, the growth of bacteria in each well was observed, and the lowest concentration that could completely inhibit the growth of bacteria was taken as the minimum inhibitory concentration (MIC) of the compound **6i**. Meanwhile, MHB broth was used as a blank control and diluted bacterial solution was used as a negative control. After reading the MIC results, the clarified wells treated with the compounds were dripped into MHA solid medium and incubated at 37°C for 24 h. The concentration of the compound to be tested corresponding to the number of colonies ≤5 was read as the minimum bactericidal concentration (MBC).

### 2.6 Hemolytic activity

5% sterile defibrillated sheep blood erythrocyte suspension was taken in a 96-well plate, and then different concentrations of **6i** solutions (0.5–128 μg/mL) were added, PBS buffer was used as the negative control and 0.1% TritonX-100 was used as the positive control, and the plate was incubated at 37°C for 1 h. After centrifugation, the supernatant was taken from the 96-well plate, and the OD_540_ value was measured by an enzyme marker. The erythrocyte hemolysis rate was calculated as follows, erythrocyte hemolysis rate = (A-A_0_)/(A_total_-A_0_) × 100%, A is the absorbance of the compounds to be tested, A_0_ is the absorbance of the negative control group, and A_total_ is the absorbance of the positive control group.

### 2.7 Time-kill kinetics assay

The plate colony counting method was used to determine the Time-Kill Kinetics test of compound **6i** against MRSA-16. Single clinical MRSA-16 colonies were cultured in 1.0 mL MHB for 16–18 h in a shaker (200 rpm, 37°C). After that, the bacterial solution was diluted 1 × 10^4^ times with MHB broth and incubated for 2.5 h to dilute it to 1 × 10^5^ CFU/mL. Different concentrations of compound **6i** (8 ×, 4 ×, 1 × MIC) were added to the bacterial broth and incubated for 8 h in a shaker (200 rpm, 37°C). The coincubation solutions at different time points (0, 0.5, 1, 2, 4, 6, and 8 h) were then appropriately diluted and applied to MHB agar plates for 24 h incubation at 37°C before colony counting. Three independent experiments were conducted in duplicate. As blank controls, no compounds were added, and vancomycin was utilized as a positive control.

### 2.8 Bacterial resistance evaluation

The MIC value increasing by more than four times above its initial value is referred to as drug resistance. Firstly, the MIC values of compound **6i** and norfloxacin against *S. aureus* ATCC 29213 were determined. Subsequently, single clones of *S. aureus* ATCC 29213 were picked into fresh MHB broth medium containing 1/2 MIC concentration of **6i**, with norfloxacin as a positive drug, and incubated at 37°C for 12 h. Then, the above bacterial solution was inoculated on the MHA culture plate containing 1/2 MIC concentration of **6i** by the three-zone streak method and incubated for 24 h. The above operations were repeated for a total of 20 generations, and the MIC values of each generation were measured by microbroth dilution method.

### 2.9 Inhibition of biofilm formation assay

Single colonies of *S. aureus* ATCC 29213 and MRSA-16 were singled out in 1 mL of TSB (containing 1% glucose, 3% NaCl) medium and incubated for 3 h at 37°C, followed by dilution to a concentration of 1 × 10^5^ CFU/mL. The compound **6i** at different concentrations (64 μg/mL, 32 μg/mL, 16 μg/mL, 8 μg/mL, 4 μg/mL, 2 μg/mL) was co-cultivated with the diluted bacterial solution for 24 h at 37°C. The medium was discarded and washed three times with sterile 0.01 M PBS solution (pH = 7.2–7.4) to remove the medium with planktonic bacteria. Then, 100 μL of anhydrous methanol was added to each well, and after fixation for 20 min, the methanol was discarded, 200 μL of 0.1% crystal violet staining solution was added, and the staining solution was left to stand for 30 min. Then the crystal violet staining solution was discarded, washed three times with PBS buffer, and air-dried. After that, 100 μL of 95% ethanol was added, and the crystal violet was dissolved on a shaker at 37°C for 30 min. The absorbance values at 570 nm of each well were measured by an enzyme marker to calculate the inhibition of *S. aureus* ATCC 29213 and MRSA-16 biofilm by different concentrations of compound **6i**. Inhibition rate = A/A_0_ (A_0_: absorbance of negative control).

### 2.10 Bacterial survival assay in biofilm

Single colonies of *S. aureus* ATCC 29213 and MRSA-16 were singled out in 1 mL of TSB (containing 1% glucose, 3% NaCl) medium and incubated for 3 h at 37°C, followed by dilution to a concentration of 1 × 10^5^ CFU/mL. Bacterial (100 μL) solution was added to a 96-well plate, followed by 100 μL of fresh TSB medium, and incubated at 37°C for 24 h. After the formation of a dense white biofilm, the medium was discarded, and the biofilm was washed three times with sterile 0.01 M PBS (pH = 7.2–7.4) to remove the medium and the planktonic bacteria. Different volumes of compound **6i** were added to the 96-well plate containing the biofilm to give a final concentration of 64 μg/mL, 32 μg/mL, 16 μg/mL, 8 μg/mL, 4 μg/mL, 2 μg/mL and cultured for another 24 h at 37°C. The medium was discarded and washed three times with sterile 0.01 M PBS solution (pH = 7.2–7.4) to remove the medium with planktonic bacteria. After disrupting the biofilm with ultrasound, the bacterial solution in the wells was diluted in a 10-fold gradient and then placed in solid medium for 16–18 h. The colonies in the solid medium were counted and the number of surviving bacteria in the biofilm after compound treatment was calculated.

### 2.11 Scanning electron microscopy

The monoclonal clone of MRSA-16 was picked in 1 mL of fresh MHB broth and incubated overnight at 37°C. Then, the bacterial solution was diluted 10-fold with MHB broth to a concentration of 1 × 10^5^ CFU/mL and then continued to incubate for 2–3 h. Compound **6i** with a final concentration of 32 μg/mL was added, and the incubation was continued for 4 h. Subsequently, centrifugation was performed, the medium was discarded, and the organisms were washed three times with 0.01 M PBS buffer (pH = 7.2–7.4). Appropriate amount of 2.5% glutaraldehyde fixative was slowly added to the EP tubes containing the organisms, and fixed overnight at 4°C for 24 h. Following that, the samples were eluted using a gradient concentration of ethanol, dried, and plated with gold at the critical point. Finally, a Hitachi Electron microscope was used to photograph the bacterial morphology.

### 2.12 Membrane depolarization assay

The monoclonal clone of MRSA-16 was picked in 1 mL of fresh MHB broth and incubated overnight at 37°C. Then, the bacterial solution was diluted 10-fold with MHB broth to a concentration of 1 × 10^5^ CFU/mL. Bacterial solution (150 μL) was placed on a 96-well bottom clear black plate, followed by 40 μL (25 μM) of DiSC3(5) fluorescent dye and incubated for 30 min at 37°C in the dark. The fluorescence intensity of the mixture was measured continuously for 10 min at 622 nm excitation and 670 nm emission wavelengths, every 2 min. After the fluorescence intensity was stabilized, 10 μL of different concentrations of **6i** (The final concentrations of 4, 8, 16, 32 μg/mL) solution were added, and the fluorescence intensity was measured every 2 min for 20 min.

### 2.13 SYTOX green assay

The monoclonal clone of MRSA-16 was picked in 1 mL of fresh MHB broth and incubated overnight at 37°C. Then, the bacterial solution was diluted 10-fold with MHB broth to a concentration of 1 × 10^5^ CFU/mL. Bacterial solution (150 μL) was placed on a 96-well bottom clear black plate, followed by 40 μL (15 μM) of SYTOX Green fluorescent dye and incubated for 30 min at 37°C in the dark. The fluorescence intensity of the mixture was measured continuously for 10 min at 500 nm excitation and 530 nm emission wavelengths, every 2 min. After the fluorescence intensity was stabilized, 10 μL of different concentrations of **6i** (The final concentrations of 4, 8, 16, 32 μg/mL) solution were added, and the fluorescence intensity was measured every 2 min for 20 min. Melittin was the positive control.

### 2.14 DAPI/PI fluorescence staining

Single colonies of MRSA-16 were incubated in MHB medium for 6 h (200 rpm, 37°C), and then **6i** was added to achieve a final concentration of 8 × MIC. Following that, the incubation was then continued for 4 h and the supernatant was removed by centrifugation for 3 min (4,000 rpm, 4°C), and MRSA-16 was resuspended in PBS. Under light-proof conditions, 20 μL of DAPI (10 μg/mL) and PI (20 μg/mL) staining solutions were pipetted into the resuspended bacterial solution, followed by 0.5 h of dark incubation. Finally, a laser confocal microscope was used to photograph fluorescence.

### 2.15 DNA leakage

MRSA-16 (1 × 10^6^ CFU/mL) was suspended in PBS, and then **6i** was added to make the final concentrations of 32, 16, 8, and 4 μg/mL, respectively, and incubated at 37°C for 4 h. After centrifugation for 4 min (3,500 rpm, 4°C), the supernatant was aspirated and the DNA concentration was determined by microspectrophotometer.

### 2.16 *In vivo* antibacterial activity

Thirty KM mice were acclimatized for 3 days and randomly divided into 5 groups (n = 6): blank group, model group, **6i** (5 mg/kg), **6i** (10 mg/kg), and vancomycin (5 mg/kg). 60 μL MRSA-16 bacterial solution (6 × 10^8^ CFU/mL) was injected subcutaneously into the back of mice, and then 60 μL compound **6i** and vancomycin were injected subcutaneously into the back of mice for treatment 1 h later. After 24 h, the mice were observed for their mental status, and the skin of the infected area on their backs was photographed. Then, mice were euthanized, and the infected skin and major organ tissues (heart, liver, spleen, lungs, and kidneys) were aseptically collected, fixed in 4% paraformaldehyde, paraffin-embedded and stained with H&E. Drip plate counts of infected skin tissues were performed to assess the antimicrobial effect of **6i**.

### 2.17 Statistical analysis

The data were all expressed as mean ± SD or mean ± SEM. SPSS 21.0 software (IBM, Inc.) was used for statistical analysis. The independent samples *t*-test or one-way ANOVA was used to compare data between two groups. Statistical significance was defined as *p*-values less than or equal to 0.05 (*p*-values, * < 0.05, ** < 0.01, *** < 0.001, **** < 0.0001).

## 3 Results

### 3.1 Synthesis of magnolol derivative **6i**

**Figure 1:**
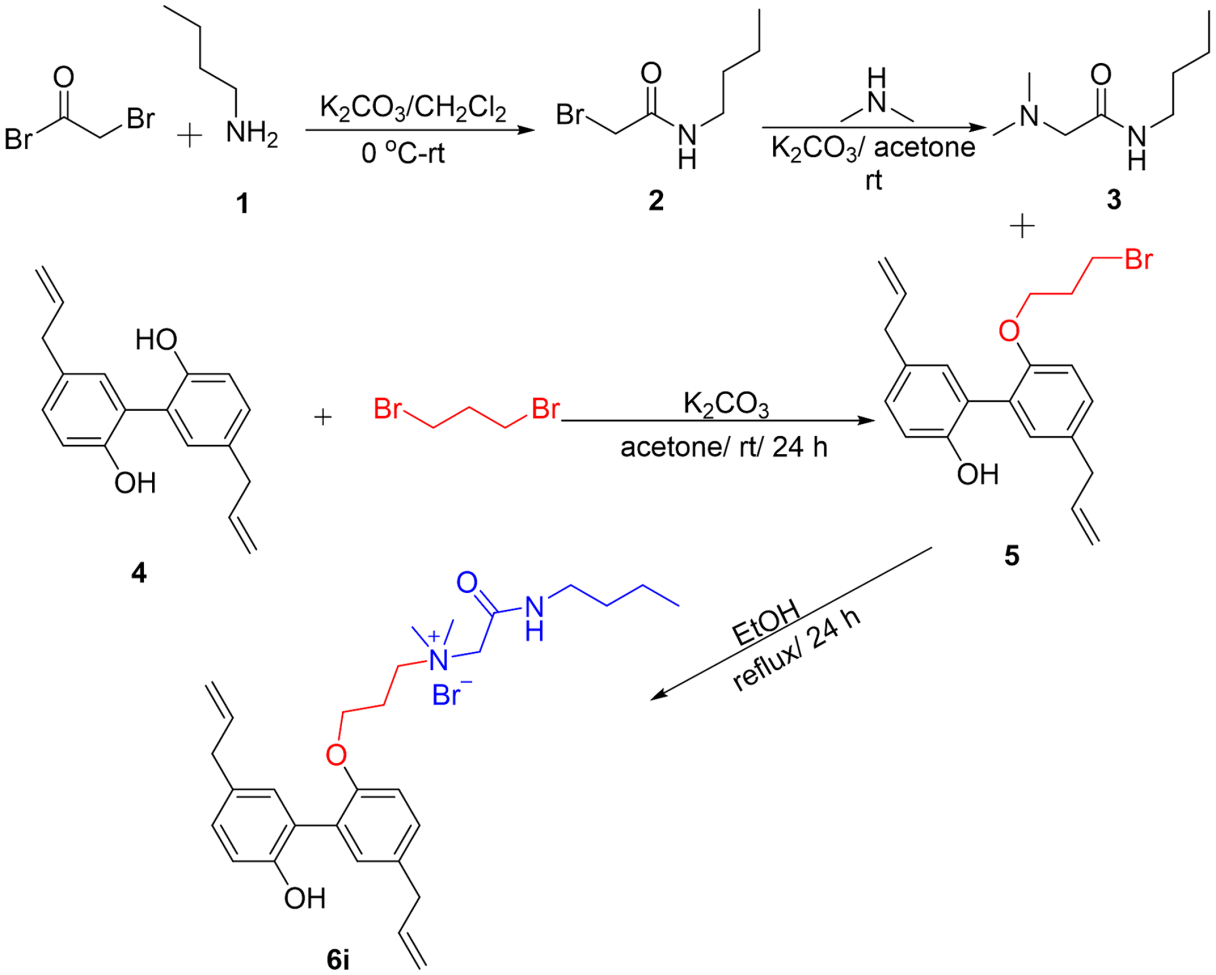
Synthesis of Magnolol Amphiphiles 6i.

The synthesis process of the magnolol derivative **6i** was detailed in Figure 1. Firstly, the reaction of bromoacetyl bromide with n-Butylamine (**1**) yielded intermediate **2**. Compound **2** was then treated with dimethylamine to obtain intermediate **3**. Subsequently, magnolol (**4**) was alkylated with 1,3-dibromopropane in the presence of K_2_CO_3_ to afford compound **5**. Finally, intermediate **5** interacted with intermediate **3** to produce magnolol derivative **6i**. The synthesized magnolol derivative **6i** were characterized by ^1^H NMR, ^13^C NMR, and HRMS. The purity of the **6i** was more than 95% by using HPLC detection. The structural characterization data of **6i** were presented below, and additional copies of spectra were provided in the Supplementary material.

*N-(2-(butylamino)-2-oxoethyl)-3-((5,5′-diallyl-2′-hydroxy-[1,1′-biphenyl]-2-yl)oxy)-N, N-dimethylpropan-1-aminium* bromide (**6i**). **6i** was prepared from compound **2B** (0.2 mmol, 77.5 mg) and intermediate **4F** (0.6 mmol) following a similar procedure as that for **5i**. Data for **6i**: Yield: 97%, pale-yellow liquid, ^1^H NMR (400 MHz, CDCl_3_) δ: 8.67 (t, J = 8.4 Hz, 1H, -NH-), 7.15 (d, J = 8.0 Hz, 1H, -Ph), 7.10 (dd, J = 8.4, 2.4 Hz, 1H, -Ph), 7.05 (d, J = 2.4 Hz, 1H, -Ph), 7.00 (dd, J = 8.0, 2.0 Hz, 1H, -Ph), 6.95 (d, J = 2.0 Hz, 1H, -Ph), 6.72 (d, J = 8.4 Hz, 1H, -Ph), 5.91–5.98 (m, 2H, -CH=CH_2_), 5.02–5.10 (m, 4H, -CH=CH_2_), 4.42 (s, 2H, -CH_2_-), 3.99 (t, J = 5.2 Hz, 2H, -CH_2_-), 3.62 (t, J = 8.0 Hz, 2H, -CH_2_-), 3.31–3.36 (m, 4H, -CH_2_-), 3.16–3.26 (m, 2H, -CH_2_-), 3.12 (s, 6H, N-CH_3_), 2.10–2.11 (m, 2H, -CH_2_-), 1.51–1.57 (m, 2H, -CH_2_-), 1.33–1.38 (m, 2H, -CH_2_-), 0.89 (t, J = 7.2 Hz, 3H, -CH_3_-); ^13^C NMR (100 MHz, CDCl_3_) δ: 162.4, 153.4, 152.2, 137.7, 137.3, 133.6, 131.8, 131.6, 131.4, 128.9, 128.8, 128.1, 125.8, 115.9, 115.5, 112.4, 64.4, 64.3, 63.8, 51.2, 51.0, 39.6, 39.3, 30.8, 23.1, 20.2, 13.6. HRMS (ESI) C_29_H_41_BrN_2_O_3_ [M-Br]^+^ calcd = 465.3112; found = 465.3120.

### 3.2 *In vitro* antibacterial and hemolytic assays

In our previous study (Guo et al., 2021a), we reported the synthesis of a series of honokiol derivatives and preliminarily verified the good antimicrobial activity of **6i**. Therefore, Magnolol derivative **6i** was evaluated for *in vitro* antimicrobial activity against *Staphylococcus aureus* ATCC 29213 (*S. aureus* ATCC 29213) and 10 clinical MRSA isolates by microbroth dilution method. Generally, when the MBC/MIC of an antibiotic is less than or equal to 4, it is considered to be an effective antibacterial agent (Biedenbach et al., 2007). The *in vitro* antimicrobial activity of **6i** against Gram-positive (G+) bacterium *S. aureus* ATCC 29213 and 10 clinical isolates of MRSA was firstly evaluated by using micro broth dilution method with reference to the standards of the Clinical and Laboratory Standards Institute (CLSI). The half maximal hemolytic concentration (HC_50_) of **6i** against sheep erythrocytes was also determined to evaluate their toxicity and membrane selectivity. As shown in Table 1, the MIC and MBC values of compound **6i** against *S. aureus* ATCC 29213 and MRSA were both 2–8 μg/mL, especially, the MBC/MIC value was 1. Meanwhile, **6i** exhibited low hemolytic activity (HC_50_ = 596.5 μg/mL) and high membrane selectivity (The ratio of HC_50_/*S. aureus* ATCC 29213 MIC, SI = 298.3). These results demonstrated that **6i** possessed good antibacterial activity and excellent membrane selectivity *in vitro*, which is worthy to be further investigated for bactericidal properties and bactericidal mechanism.

**Table 1:** The MIC and MBC values of compound **6i** to *S. aureus* ATCC 29213 and 10 clinical MRSA isolates as well as the values of the HC_50_ (μg/mL) and SI.

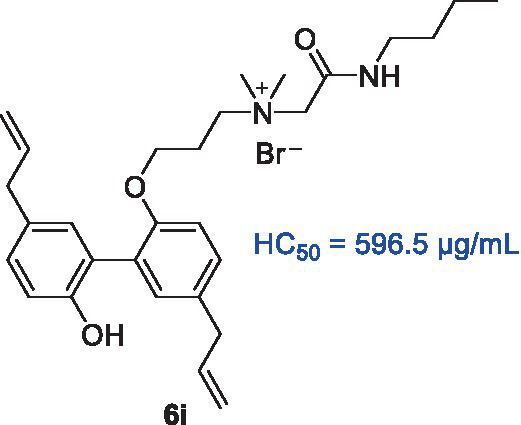				
Bacterial strain	MIC (μg/mL)	MBC (μg/mL)	MBC/MIC	SI^a^
*S. aureus*	2	2	1	298.3
MRSA-11	8	8	1	74.6
MRSA-12	4	4	1	149.2
MRSA-13	4	4	1	149.2
MRSA-14	4	4	1	149.2
MRSA-15	4	4	1	149.2
MRSA-16	4	4	1	149.2
MRSA-17	4	4	1	149.2
MRSA-18	4	4	1	149.2
MRSA-19	4	4	1	149.2
MRSA-20	4	4	1	149.2

a SI: Selectivity Index (HC_50_/MICs). The experiment was repeated three times.

### 3.3 Time-kill kinetics and resistance development studies

**Figure 2:**
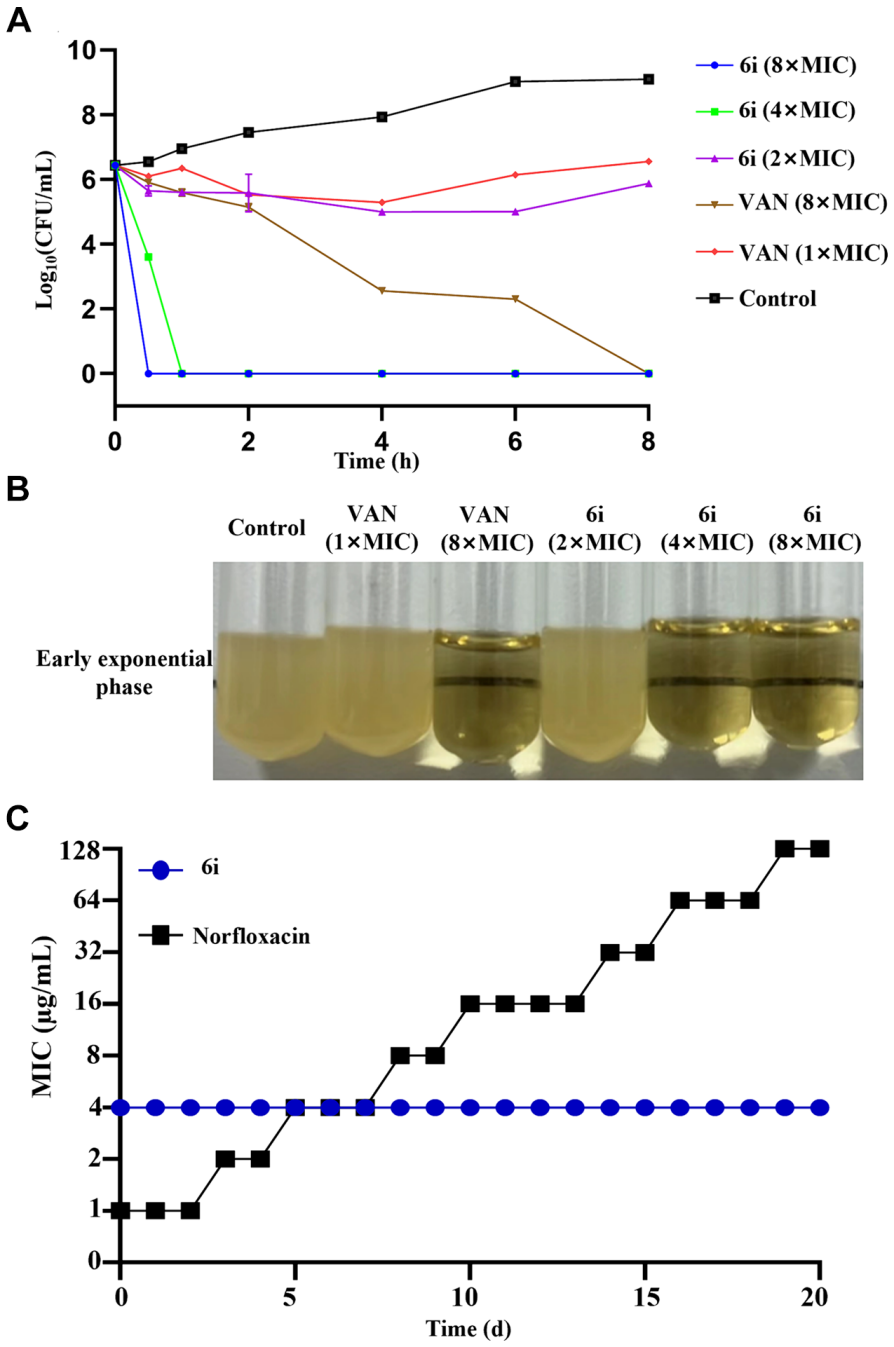
**(A)** Time-kill kinetics of compound 6i (8 ×, 4 ×, 2 × MIC, i.e., 32, 16, 8 μg/mL) against MRSA-16 at early exponential phase. **(B)** Bactericidal effect of compound 6i (8 ×, 4 ×, 2 × MIC, i.e., 32, 16, 8 μg/mL) after treatment of MRSA-16 at early exponential phase for 8 h. **(C)** Bacterial resistance study of 6i against MRSA-16. The experiment was repeated three times.

In order to further investigate the bactericidal properties of compound **6i**, vancomycin, the drug of the last line of defense for MRSA (Liu et al., 2021), was used as a positive drug, and different concentrations (8 ×, 4 ×, 2 × MIC, i.e., 32, 16, 8 μg/mL) of **6i** were determined for dynamic bactericidal efficiency of MRSA-16 at the early exponential phase. As illustrated in Figure 2A, **6i** exhibited a dose-dependent bactericidal properties and a rapid bactericidal effect against MRSA-16 at early exponential phase. Specifically, **6i** could completely kill MRSA-16 at the early exponential phase within 1 h at a concentration of 8 × MIC and within 2 h at a concentration of 4 × MIC. In contrast, vancomycin achieved complete bactericidal effect at 8 h. Moreover, from the turbidity diagram of the bacterial fluids of each group at 8 h (Figure 2B), it can be seen that for MRSA-16 bacteria at the early exponential phase, the bacterial fluids of **6i** (4 × and 8 × MIC) and vancomycin (8 × MIC) groups were clarified and transparent compared with the control group. These results demonstrated that **6i** had a strong and rapid bactericidal effect on MRSA-16.

With the increasing problem of bacterial resistance, the ability to resist bacterial resistance has become one of the important factors to evaluate the development potential of new antibacterial agents (Farrell et al., 2011; Guo et al., 2021b). Studies have reported that when the MIC value of an antibiotic increases by four times against bacteria, it is generally considered that the bacteria has developed resistance to the antibiotic (Farrell et al., 2011). Norfloxacin, as a quinolone, targets bacterial DNA topoisomerase, which is susceptible to point mutations that mediate high levels of resistance when induced by short-term drug action or sub-therapeutic doses. Therefore, the use of Norfloxacin as a reference drug makes it easy to compare whether the compounds to be tested are susceptible to drug resistance at sub-concentration doses and short-term action times. In order to investigate whether **6i** possessed low-frequency resistance, the change in the MIC value of **6i** was examined during 20 passages of successive stimulation of *S. aureus* ATCC 29213 with sub-MIC (1/2 MIC) concentrations of **6i**. After 20 consecutive generations of stimulation, the MIC value of **6i** against *S. aureus* ATCC 29213 did not show any change and remained at 4 μg/mL (Figure 2C), whereas in the norfloxacin group, the MIC value against *S. aureus* ATCC 29213 had already increased 4-fold by generation 5, and the MIC value increased 128-fold by generation 20. These results indicated that **6i** has rapid bactericidal ability and was less likely to induce resistance in bacteria.

### 3.4 Bacterial biofilm disruption study

**Figure 3:**
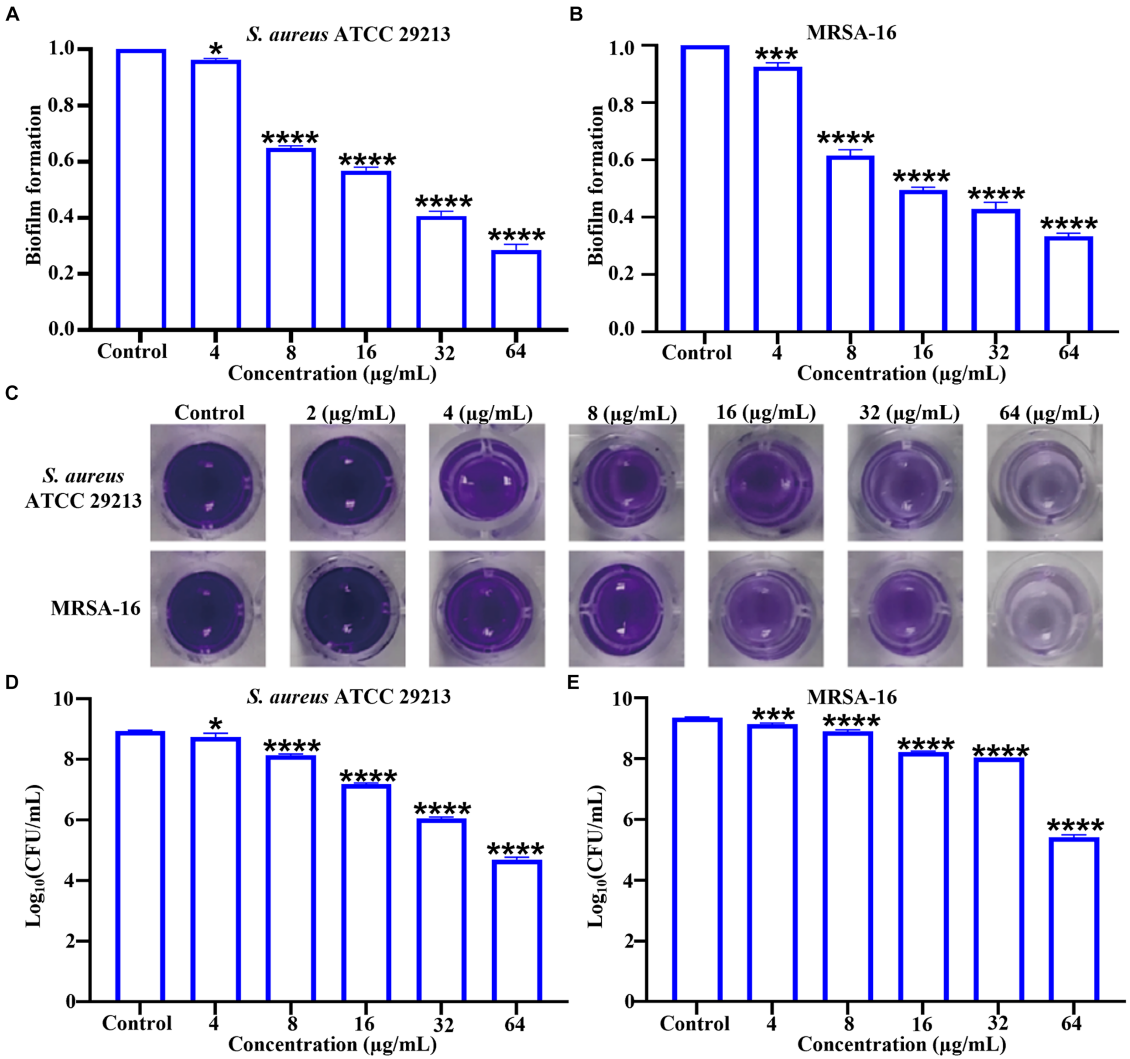
Inhibitory effect of compound 6i on bacterial biofilms. **(A,B)** The inhibitory effect of compound 6i on the biofilm formation of *S. aureus* ATCC 29213 and MRSA-16. **(C)** The crystal violet staining results of *S. aureus* ATCC 29213 and MRSA-16 biofilms treated with compound 6i. **(D,E)** The effect of compound 6i on the viable bacteria in the biofilm of *S. aureus* ATCC 29213 and MRSA-16. *p < 0.05, ***p < 0.001, ****p < 0.0001, compared with control group. Data are expressed as the mean ± SD. Error bars are representatives of three independent experiments. *p*-values were calculated using ordinary one-way ANOVA.

Biofilm formation is a universal property of bacteria (López et al., 2010). Biofilms are microbial membranous structures formed by the extracellular matrix produced by bacteria wrapped around the bacterial community, which increases the resistance of the bacteria within the membrane to exogenous stresses (such as antibiotics and immunological substances.) and allows the bacteria to survive in unfavorable environments (Gunn et al., 2016). Crystalline violet staining was used to investigate the ability of **6i** to inhibit biofilm formation. As can be seen in Figures 3A–C, compound **6i** effectively inhibited the formation of biofilms in both *S. aureus* ATCC 29213 and MRSA-16 in a dose-dependent manner. Concurrently, Figures 3D,E illustrated that at a concentration of 64 μg/mL, compound **6i** significantly reduced the viable counts in the biofilms of *S. aureus* ATCC 29213 and MRSA-16 by 1.89 log CFU/mL and 1.72 log CFU/mL, respectively, compared to the initial counts. These results demonstrated that **6i** could dose-dependently inhibit the formation of bacterial biofilm and had a killing effect on the viable bacteria within the bacterial biofilm.

### 3.5 Antimicrobial mechanism studies

#### 3.5.1 Scanning electron microscopy

**Figure 4:**
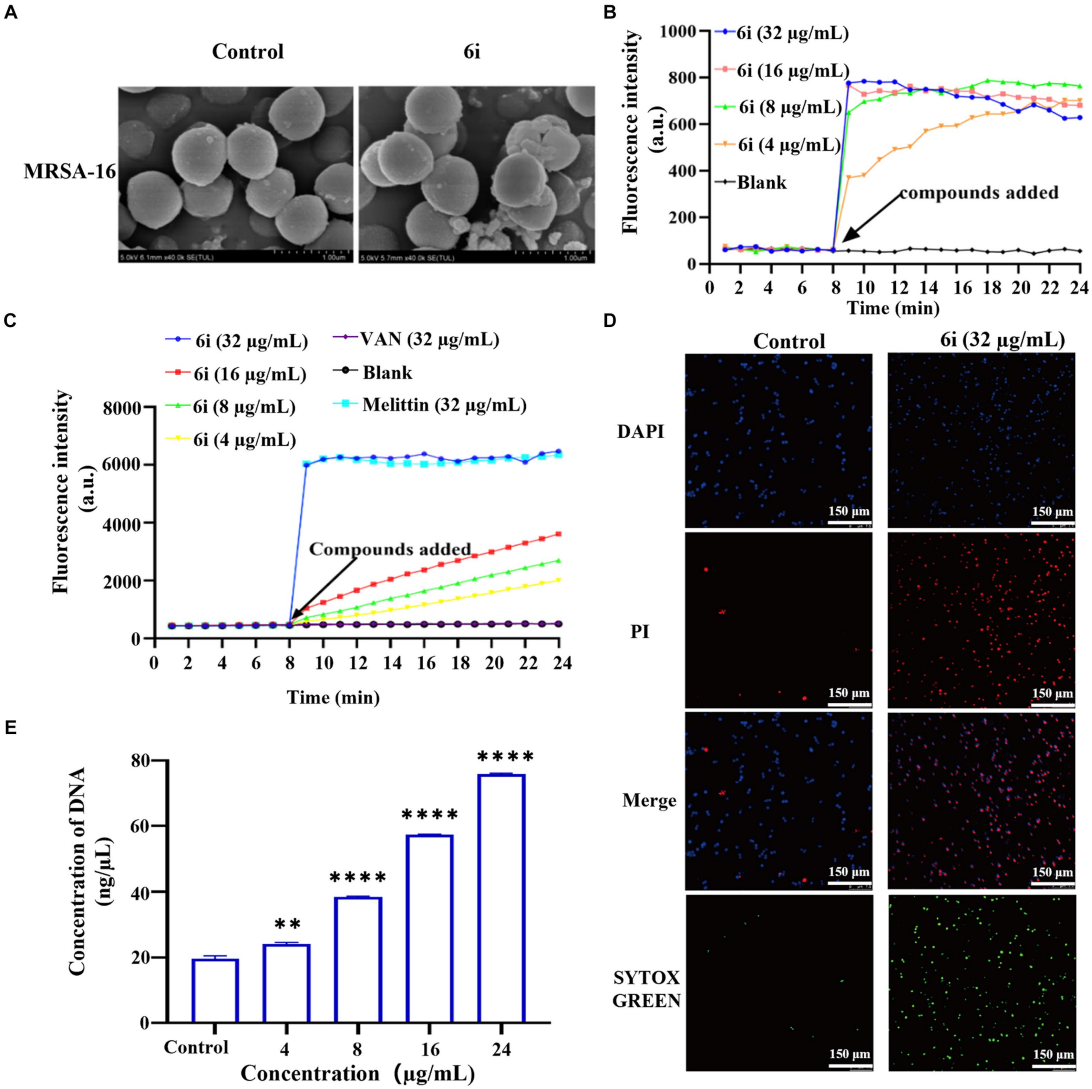
Antibacterial mechanism of compounds 6i against the clinical MRSA-16 isolate. **(A)** SEM images of the cell membrane of MRSA-16 cells. MRSA-16 cells without compound treatment (control); Scale bar: 1.0 μm. **(B,C)** Cytoplasmic membrane depolarization of 6i using DiSC3(5) and SYTOX Green assays, respectively. The blank control was bacteria without drug treatment, and melittin was used as a positive control. **(D)** Fluorescence micrographs of MRSA-16 cells after 6i-treated stained with DAPI, PI and SYTOX Green. Control: no treatment. Scale bar 50 μm. **(E)** DNA leakage caused by the action of compound 6i on MRSA-16. **p < 0.01, ****p < 0.0001, compared with control group. Data are expressed as the mean ± SD. Error bars are representatives of three independent experiments.

Time-kill kinetics results showed that **6i** could completely kill bacteria within 1 h under the concentration of 32 μg/mL. To further observe the damage effect of **6i** on bacterial cell membrane more directly, the 32 μg/mL of 6i was selected to treat MRSA-16 for SEM observation. As shown in Figure 4A, the surface of MRSA-16 cell membrane in the blank group was intact and smooth. While after treatment with **6i**, the surface of MRSA-16 cell membrane underwent an increase in roughness and had particulate matter, some of the bacteria were broken, and adhesion between the bacteria occurred. These results indicated that **6i** could disrupt the bacterial cell membrane, exerting a bactericidal effect.

#### 3.5.2 Membrane depolarization and permeabilization assay

When the antibacterial compound acted on the bacterial cell membrane, a series of physiological activities and functions relying on the cell membrane were correspondingly affected and altered (Koh et al., 2015). Firstly, DiSC3(5) (3,3′-dipropylthiadicarbocyanine iodide) was used to analyze whether **6i** could induce depolarization of the cell membrane in MRSA-16. DiSC3(5) is a membrane potential-sensitive probe that accumulates within the phospholipid bilayer of bacterial cell membranes, causing self-quenching of DiSC3(5) fluorescence. When the bacterial cell membrane undergoes depolarization, resulting in the loss of membrane potential, DiSC3(5) is released into the solution, causing an increase in fluorescence intensity. As shown in Figure 4B, compound **6i** could dose-dependently enhance the fluorescence intensity of DiSC3(5). When the concentration of **6i** was 4 and 16 μg/mL, the fluorescence intensity increased by 5 and 10 times, respectively, compared to the blank group. Subsequently, SYTOX Green dye was utilized to assess the impact of **6i** on cell membrane permeability. Melittin is a natural AMP, and its antibacterial target is the cell membrane, which can cause changes in the permeability of the cell membrane. Vancomycin can achieve the antibacterial purpose by inhibiting the synthesis of the cell wall. Therefore, melittin was used as a positive control drug and vancomycin as a negative control drug. From Figure 4C, compound **6i** could dose-dependently increase the fluorescence intensity of SYTOX Green, and the fluorescence intensity at a concentration of 32 μg/mL rapidly increased 15-fold within 1 min, which was almost the same as that of the positive control, melittin, a membrane-targeting antibacterial peptide. In contrast, the SYTOX Green dye fluorescence intensity in the vancomycin group showed no change compared to the blank control. This indicates that the mechanism of action of **6i** is similar to that of melittin, which could rapidly cause changes in the permeability of the bacterial cell membrane. These findings illustrated that **6i** could target the bacterial cell membrane, inducing changes in membrane permeability and depolarization of the cell membrane, ultimately leading to bacterial death.

#### 3.5.3 Fluorescence microscopy

In order to further verify the effect of **6i** on the bacterial cell membrane, DAPI (4′,6-diamidino-2-phenylindole) and PI (propidium iodide) were used to investigate the integrity of the cell membrane of MRSA-16 after treatment with **6i** (consistent with the concentration of **6i** in SEM experiment). The DAPI dye was able to penetrate through all the cell membranes, and emitted a strong green fluorescence when it bound to the DNA, while the PI dye did not have any cell membrane permeability, and was not able to penetrate through the cell membranes of living cells, but only through the dead cells and bound to the DNA to emit a red fluorescence. Compared with the control group (Figure 4D), the red fluorescence signal of MRSA-16 group after **6i** treatment increased significantly. The result showed that **6i** could disrupt the cell membrane of MRSA-16, allowing PI to enter the cell and bind with DNA, emitting red fluorescence.

#### 3.5.4 Leakage of DNA

Once the integrity of the bacterial cell membrane is disrupted, the efflux of bacterial contents and the influx of external substances increase the vulnerability of the bacteria, leading to bacterial death (Trombetta et al., 2005). As shown in Figure 4E, compound **6i** could dose-dependently increase the concentration of DNA in MRSA-16 supernatant. Compared to the blank control group, **6i**-treated MRSA-16 showed significant leakage of DNA. These findings confirmed that compound **6i** could effectively target and disrupt the bacterial cell membrane, leading to increased DNA leakage and consequently causing bacterial death.

### 3.6 Plasma stability

Upon entering the bloodstream, antimicrobial agents are subject to the influence of plasma proteins and various hydrolytic enzymes, resulting in a reduction of their effective concentrations within the body. Hence, plasma stability stands is one of the key factors impacting the pharmaceutical development of antimicrobial compounds (Soheili et al., 2019). As illustrated in Supplementary Figure S4A, compared to the conditions in the MHB medium, the antimicrobial efficacy of **6i** against MRSA-16 experienced a certain degree of attenuation in a 50% plasma environment, with the MBC value increasing from 2 to 4 μg/mL. However, the MBC value of **6i** remained consistently stable within 6 h. This result suggested that the antimicrobial effect of **6i** was affected to some extent in the plasma but could still maintain a robust and prolonged *in vivo* antimicrobial effect (Supplementary Figure S4).

**Table 2:** *In vivo* toxicity of compound **6i.**

Dose (mg/kg/d)	Number	Subcutaneous injection
		Survival rate (%)
Control	6	100%
80	6	100%
40	6	100%
20	6	100%
10	6	100%
5	6	100%

### 3.7 Safety evaluation

In order to evaluate the safety of **6i**, we first selected mouse embryonic fibroblasts (3 T3) for cytotoxicity testing. As shown in Supplementary Figure S4B, at a concentration of 32 μg/mL of compound **6i**, the cell viability remained above 80%. This result proved that **6i** exhibited low toxicity to normal cells, with a safe concentration significantly exceeding its effective antimicrobial concentration. Further, *in vivo* safety results showed that that **6i** still guaranteed 100% survival at doses ranging from 5-80 mg/kg/d (Table 2). Subsequently, mice in the **6i** (10 mg/kg) group were euthanized, and blood was collected for further hematological and biochemical analysis. Simultaneously, skin at the injection sites and major organ tissues were subjected to H&E staining. The results indicated that in comparison to the blank control group, mice in the **6i** (10 mg/kg) did not show any abnormalities in hematological and biochemical parameters (Supplementary Figure S5). H&E staining further confirmed the absence of pathological changes in the skin at the injection sites and major organs of mice in **6i** (10 mg/kg) group (Supplementary Figure S6). These findings indicated that **6i** exhibited good *in vivo* safety, and 10 mg/kg could be considered a safe dosage for further evaluation of its *in vivo* antimicrobial activity.

### 3.8 *In vivo* antibacterial assay

**Figure 5:**
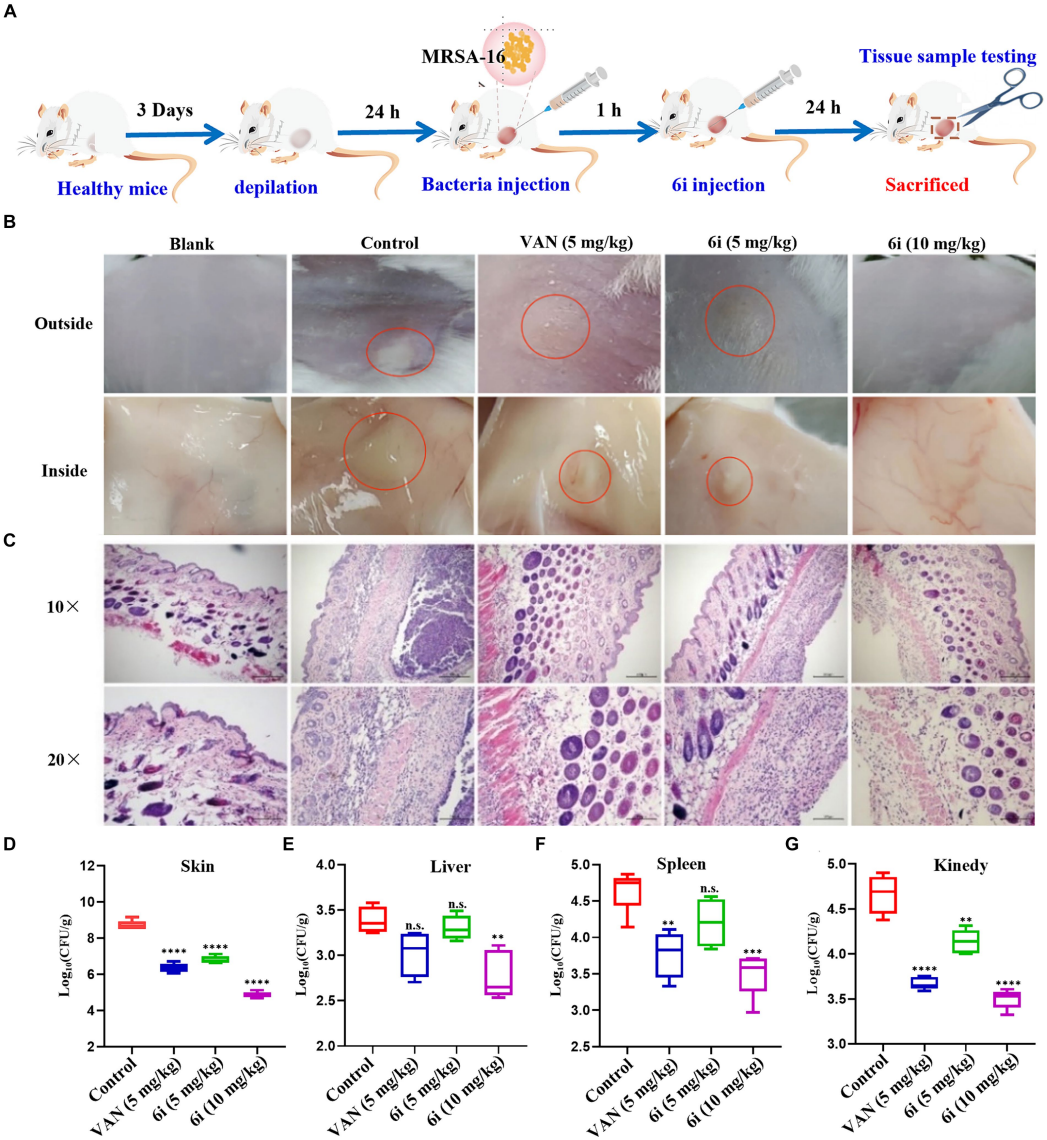
*In vivo* antibacterial assay. **(A)** Schematic of the experimental protocol for the murine abscess model of MRSA infections. **(B)** Photographs of MRSA infection sites with various treatments 24 h after infection. **(C)** Histological photographs of infected skin that received various treatments. Scale bar is 200 or 100 μm. **(D–G)** Bacterial survival in the skin, liver, spleen, kidney of MRSA-infected mice of each group treated with 6i (5 mg/kg), 6i (10 mg/kg), and vancomycin (Van) (5 mg/kg) for 24 h. The data are presented as the means ± SE from six independent experiments, **p < 0.01, ****p < 0.0001 (determined by an ordinary one-way ANOVA).

The antimicrobial effect of **6i** *in vivo* was investigated by constructing a mouse model of MRSA-16 subcutaneous abscess infection (Figure 5A). As shown in Figure 5B, the control group mice exhibited evident abscesses and inflammation in the back skin and subcutaneous tissues. In comparison, the symptoms in the mice treated with 5 mg/kg of **6i** and the vancomycin (5 mg/kg) group were significantly alleviated. Notably, mice treated with **6i** (10 mg/kg) showed no observable abnormalities, similar to the blank group. We also observed the bacterial load and H&E staining results for the liver, spleen, and kidneys. H&E staining results (Figure 5C) revealed a substantial presence of scattered inflammatory cell infiltration in the subcutaneous tissues of model mice, manifesting as purulent infection infiltrating the epidermis and dermis. In the vancomycin group (5 mg/kg) and the **6i** group (5 mg/kg), mice exhibited scattered inflammatory cell infiltration in the skin, showing slight improvement compared to the model group. Notably, mice treated with **6i** (10 mg/kg) demonstrated a significant improvement in skin inflammation, with only minimal scattered inflammatory cell infiltration observed. Subsequently, aseptic dissection was performed to collect the infected sites of mice from each group for bacterial plate colony counting. The counting results revealed a significant reduction in tissue bacterial load for all treatment groups compared to the control group. Specifically, the skin bacterial load decreased by 1.3 log CFU/g in the **6i** (5 mg/kg) group, 1.5 log CFU/g in the vancomycin (5 mg/kg) group, and 1.8 log CFU/g in the compound **6i** (10 mg/kg) group (Figures 5D–G).

Additionally, H&E staining results for the liver, spleen, and kidneys in each treatment group showed no pathological changes. It is speculated that the primary infection site of the bacteria is the skin, forming a subcutaneous abscess. The bacterial loads in the liver, spleen, and kidneys did not reach levels that would induce organ pathology (Supplementary Figure S7). These results indicate that compound **6i** exhibited a robust antibacterial effect *in vivo*, and its efficacy is comparable to vancomycin at equivalent doses.

## 4 Discussion

Among the common clinical MDR, methicillin-resistant *Staphylococcus aureus* (MRSA) is an important pathogen causing hospital-acquired and community-acquired infections, and even to death (Mun et al., 2022). More important is, the clinical drugs currently in use against MRSA, including vancomycin, daptomycin, and linezolid, have encountered varying degrees of resistance due to the emergence and spread of corresponding resistance genes. This has placed the treatment of MRSA infections in a nearly untenable position with limited effective therapeutic options available (Darby et al., 2023; Hou et al., 2023). Therefore, there is an urgent need to develop new anti-MRSA drugs with new molecular entities or new modes of action. Compared to conventional antibiotics, natural products have attracted greater attention because of their easy access to sources, low cost, pharmacological activity, and minimal side effects.

Based on the amphiphilic structural characteristics of AMP, our group initially induced AMP mimics into the 2, 4′-OH site of magnolol resulting in bis-substituted magnolol derivatives **5a-r**, and the single 4′-OH substituted magnolol derivatives were also synthesized as **6a-r**. As **5i** was screened out as the most effective antimicrobial compound and its antimicrobial mechanisms and *in vitro* and *in vivo* activities have been comprehensively explored (Guo et al., 2021a). However, it is not clear whether single-substituted magnolol derivatives have the same antibacterial properties as double-substituted magnolol derivatives *in vitro* and *in vivo*. As **5i** and **6i** share the same AMP mimetic fragment and linker, and both exhibit good antibacterial activity, low hemolytic activity and high membrane selectivity. In this study, we systematically explored the antibacterial properties and antibacterial mechanisms of **6i** *in vitro* and *in vivo*. We found that **6i** also exhibits faster time-killing dynamics. At the concentration of 8 × MIC, **6i** could completely kill MRSA within 0.5 h, representing a fast and stable sterilizing effect. Moreover, compound **6i** did not induce drug resistance under subMIC induction for 20 generations, and could inhibit the biofilm formation and significantly kill the bacteria in the biofilm at high concentrations. The stable mutation rate of bacterial cell membrane structure is low, which is an ideal antibacterial target that is not easily cause drug resistance. AMP has strong cationic characteristics and can interact with negatively charged bacterial cell membranes, showing strong antibacterial activity and high selectivity (Mookherjee et al., 2020; Portelinha et al., 2021). The structural design of **6i** was inspired by AMP, which is a new antimicrobial chemical entity containing the natural product magnolol, linker, and AMP mimetic. The lipophilic structure Magnolol facilitates the insertion of magnolol amphiphiles into bacterial phospholipid bilayers, while AMP mimetic fragments perform as hydrophilic cationic moieties to improve water solubility and destroy bacterial cell membranes through electrostatic interactions, eventually leading to bacterial death. Mechanistic studies showed that compound 6i exhibited the same bacterial cell membrane-targeted antibacterial mechanism as **5i**, that is, the single substituted magnolol derivatives also successfully mimic the amphiphilic structure and bacterial cell membrane-targeted antibacterial properties of AMP. In addition, **6i** was also shown to have good plasma stability and safety *in vitro* and *in vivo*, which are key factors for the antibacterial effect *in vivo*. In a mouse subcutaneous abscess infection model, 6i also showed promising *in vivo* anti-infective potency. Unfortunately, although **6i** showed similar antibacterial effects against the infection in the skin, liver, and spleen of mice at the same dose, its antibacterial activity in the kidney was not as good as that of the positive control vancomycin. This may be due to its smaller molecular weight and good biodegradable amide bond compared with the macromolecular glycopeptide component vancomycin, which allows **6i** to biodegrade before it enters the kidney and accumulates, thus failing to maintain an effective antimicrobial concentration in the kidney. However, from another perspective, these features also ensure good *in vivo* safety of **6i**, with the advantage of being less likely to develop drug resistance.

## 5 Conclusion

To sum up, the magnolol derivative **6i**, characterized by its amphiphilic structure akin to antimicrobial peptides, demonstrates exceptional activity against *Staphylococcus aureus* ATCC29213 and clinical MRSA isolates (MICs = 2–8 μg/mL), low hemolytic activity (HC_50_ = 596.5 μg/mL) and membrane selectivity (SI = 298.3). Compound **6i** exhibited faster bactericidal efficiency than conventional antibiotics, completely eliminating both early and late exponential phase of MRSA within 0.5 and 2 h at a concentration of 8 × MIC (32 μg/mL), respectively, and had a lower propensity for inducing bacterial resistance. **6i** also could inhibit bacterial biofilm formation and kill bacteria within biofilms in a dose-dependent manner. Mechanistic studies further revealed that **6i** targeted bacterial cell membranes, causing depolarization and increased permeability, leading to membrane integrity disruption and DNA leakage, resulting in bacterial death. Meanwhile, **6i** also showed good plasma stability and excellent *in vitro* and *in vivo* safety. In a mouse skin abscess model of MRSA-16 infection, **6i** demonstrated potent *in vivo* antimicrobial activity, comparable to vancomycin. These findings suggested that the magnolol derivative **6i** had potential as a novel anti-MRSA agent.

## Data availability statement

The original contributions presented in the study are included in the article/Supplementary material, further inquiries can be directed to the corresponding authors.

## Ethics statement

The animal study was approved by the Animal Protection and Utilization Committee of Zhengzhou University, approval number: SCXK(Jing)-2021-0006. The study was conducted in accordance with the local legislation and institutional requirements.

## Author contributions

FZ: Conceptualization, Data curation, Project administration, Writing – review & editing. HF: Data curation, Formal analysis, Validation, Visualization, Writing – review & editing. YZ: Validation, Writing – review & editing. BZ: Validation, Writing – original draft. SQ: Conceptualization, Resources, Writing – review & editing. YW: Validation, Writing – original draft. YG: Conceptualization, Supervision, Writing – review & editing. JL: Conceptualization, Funding acquisition, Project administration, Writing – original draft, Writing – review & editing. TX: Conceptualization, Formal analysis, Writing – original draft.
