# Supplementary material for: A membrane-targeting magnolol derivative for the treatment of methicillin-resistant Staphylococcus aureus infections
Source: Front Microbiol. 2024 May 17;15:1385585. doi: 10.3389/fmicb.2024.1385585 (PMC11140843; doi:10.3389/fmicb.2024.1385585)
Supplement: Supplementary file 1 [file Data_Sheet_1.doc]

**Supplementary Materials**

**A Membrane-Targeting Magnolol Derivative for the Treatment of Methicillin-Resistant *Staphylococcus Aureus* (MRSA) Infections**

Fushan Zhang^1*^, Hui Fang^2^, Yuxin Zhao^2^, Buhui Zhao^2^, Shangshang Qin, Yu Wang, Yong Guo^2,3*^, Jifeng Liu^2*^, Ting Xu^3*^

*^1^Department of Clinical Laboratory, The First Affiliated Hospital of Zhengzhou University, Zhengzhou, Henan, China;*

*^2^* *School of Pharmaceutical Sciences, Zhengzhou University, Zhengzhou, Henan, China; Key Laboratory of Advanced Drug Preparation Technologies, Ministry of Education, Zhengzhou University, Zhengzhou, Henan, China;*

*^3^Hunan Province Cooperative Innovation Center for Molecular Target New Drug Study, School of Pharmaceutical Science, Hengyang Medical School, University of South China, Hengyang, 421001, Hunan Province, China*

***Corresponding Authors**

E-mail: Fushan Zhang (Email: zhfsh01@163.com), Yong Guo (guoyong_122@163.com), Jifeng Liu (Email: Liujf2009y@126.com), Ting Xu (Email: 18337173539@163.com)

1. **Plasma stability assay.**

The supernatant was obtained as freshly prepared plasma after centrifuging sterile sheep blood at 3500 rpm for 10 min. Different concentrations of **6i** were co-incubated with 50 % plasma in a 96-well plate, and 150 *μ*L of bacterial suspension was added at different time points (0, 3 and 6 h) to continue the incubation at 37 °C for 16-24 h. After reading the MIC values, the MBC was continued to be determined. The stability of the **6i**'s antimicrobial activity in plasma was evaluated by comparing the changes in MBC at different time points.

1. **MTT Cytotoxicity Assay.**

The cytotoxicity of **6i** against mouse embryonic fibroblasts 3T3 was assessed by using the MTT (3-(4,5-dimethylthiazol-2-yl)-2,5-diphenyltetrazolium bromide) assay. 3T3 cells were inoculated at a density of 5 × 10^3^/well in 96-well plates at 37 ℃ with 5% CO_2_ for 24 h. The cells were then treated with different concentrations of **6i** (2-64 *µ*g/mL) for 24 h. Subsequently, 10 *µ*L of MTT (5.0 mg/mL) solution was added sequentially and incubated for another 4 h. The supernatant was discarded, and each well received 100 *µ*L of DMSO. After the thyroxine had completely dissolved, the absorbance of each well was measured at 570 nm with a microplate reader (M200 pro, Switzerland).

1. ***In Vivo* Toxicity.**

Healthy female MK mice at the age of 4−6 weeks (17-24 g) were purchased from Zhengzhou University Laboratory Animal Center. All animal experiments were reviewed and carried out in accordance with the “Institutional Animal Care and User Committee guidelines’ of Zhengzhou University. The mice were randomly divided into 6 groups (n=5), and treated with normal saline (control), 5, 10, 20, 40, and 80 mg/kg **6i** *via* hypodermic injection. After 24 h, the status of each group of mice was observed and recorded, as well as the skin condition of the injection site (with or without symptoms of redness, swelling, hardening, and ulceration). Finally, the mice were executed and blood was collected for routine blood and blood biochemical tests. At the same time, skin at the injection site, heart, liver, spleen, lungs, kidneys of mice were collected for H&E staining.

**4.** **Spectral Data for Compound6i**


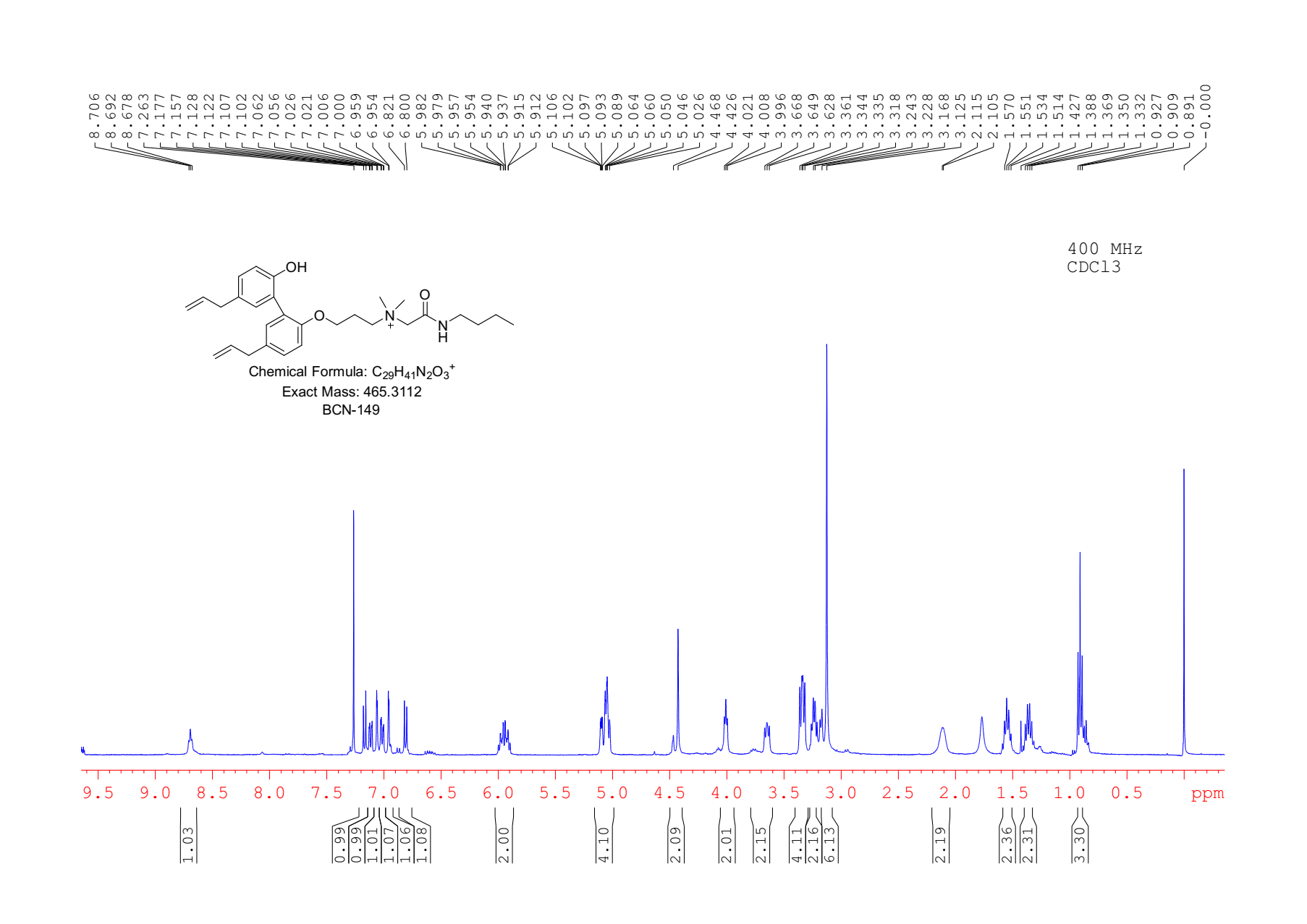


**Figure S1.** ^1^H NMR spectrum of **6i**


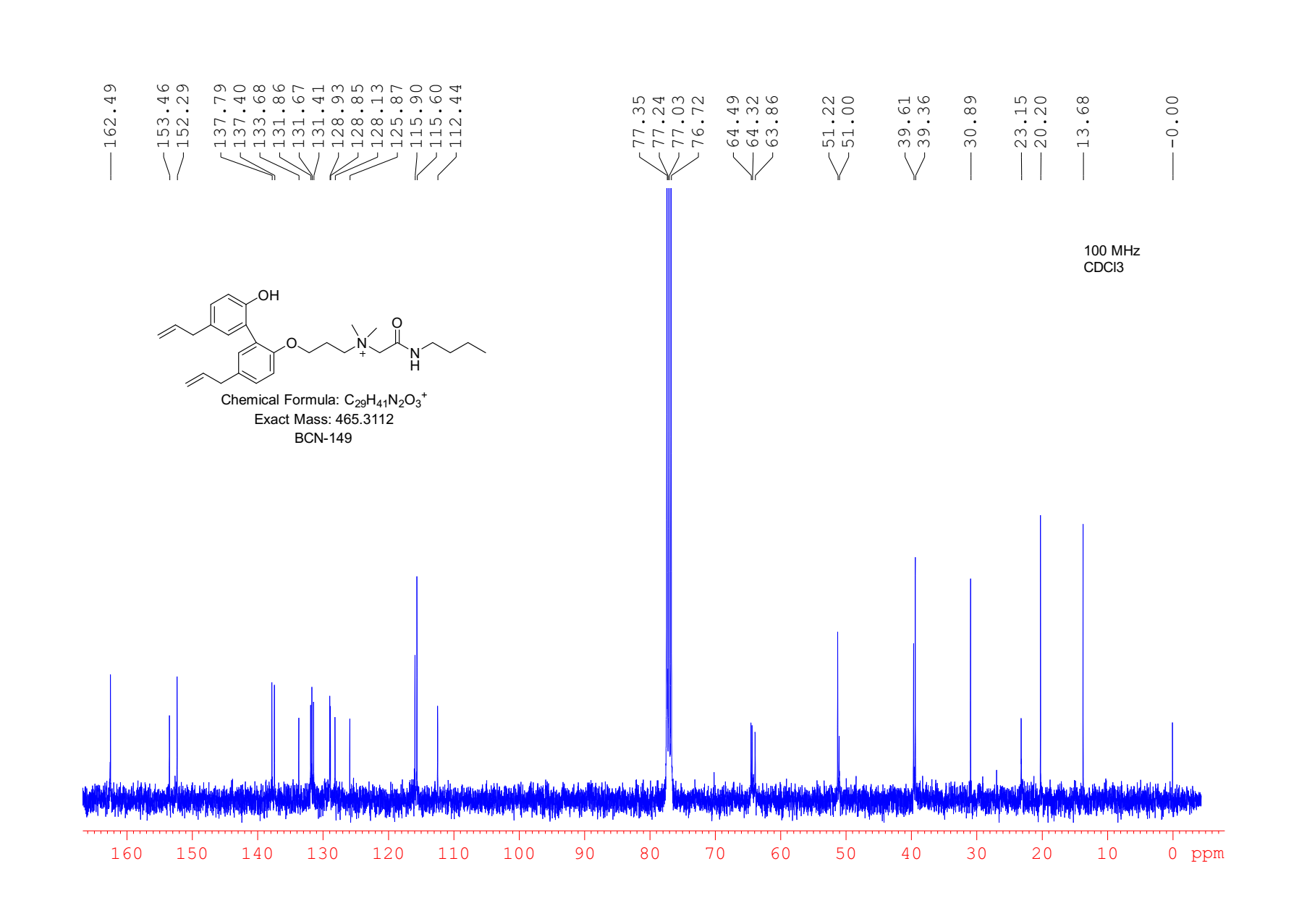


**Figure S2.** ^13^C NMR spectrum of **6i**

**
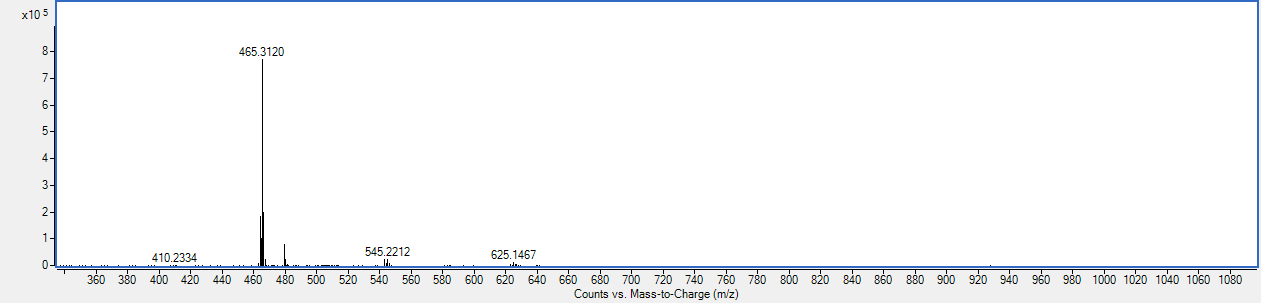
**

C_29_H_41_BrN_2_O_3_ [M-Br]^+^ calcd = 465.3112; found = 465.3120.

**Figure S3.** HRMS spectrum of **6i**

1. **Plasma stability and cytotoxicity of compound 6i**

**
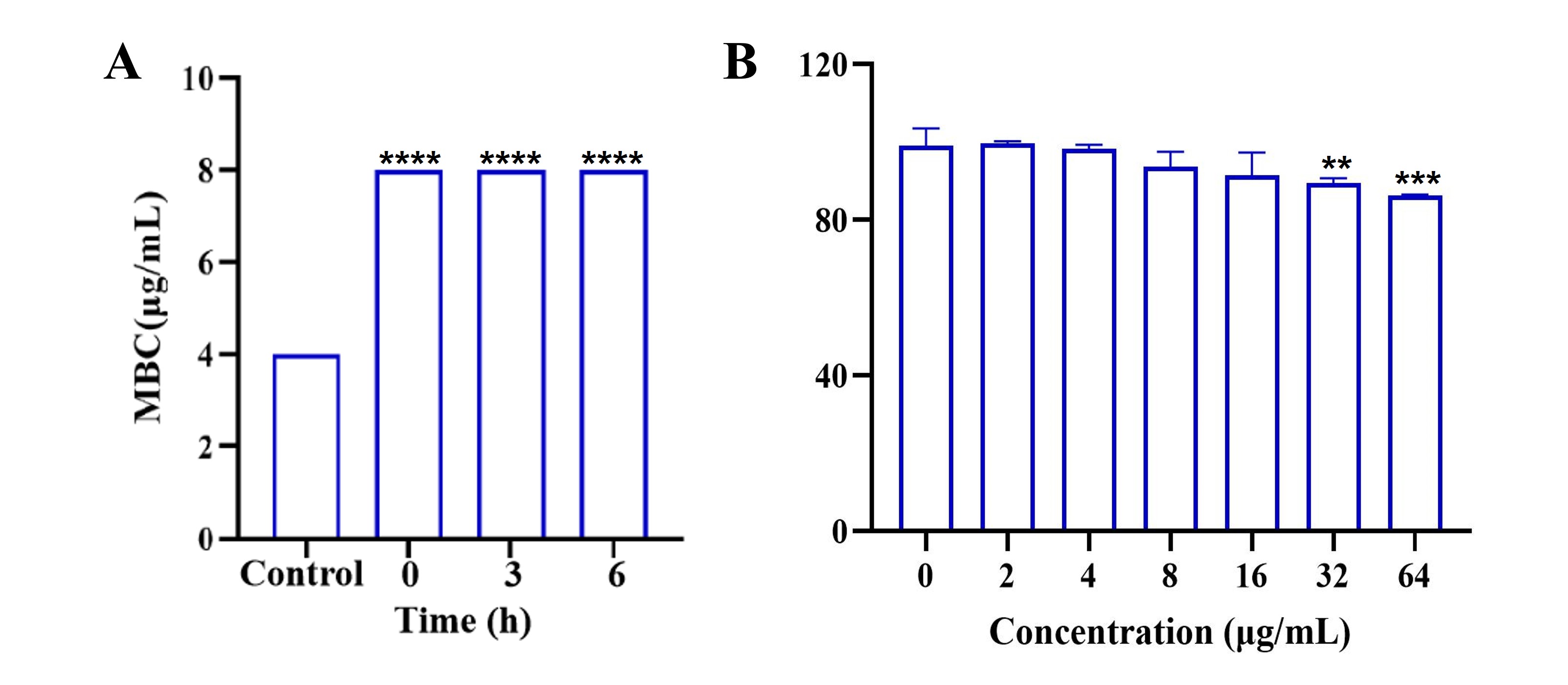
**

**Figure S4.** Plasma stability and cytotoxicity of compound **6i**. (A) MBC values of compound **6i** against MRSA-16 after 0 h, 3 h and 6 h incubation in 50 % plasma. (B) Cell viability of 3T3 cells treated with the indicated concentrations of compound 6i (2, 4, 8, 16, 32 µg/mL) for 24 h. **P < 0.01, ***P < 0.001, ****P < 0.0001, compared with control group. Data are expressed as the mean ± SD. Error bars are representatives of three independent experiments. P-values were calculated using ordinary one-way ANOVA.

1. ***In Vivo* Toxicity.**


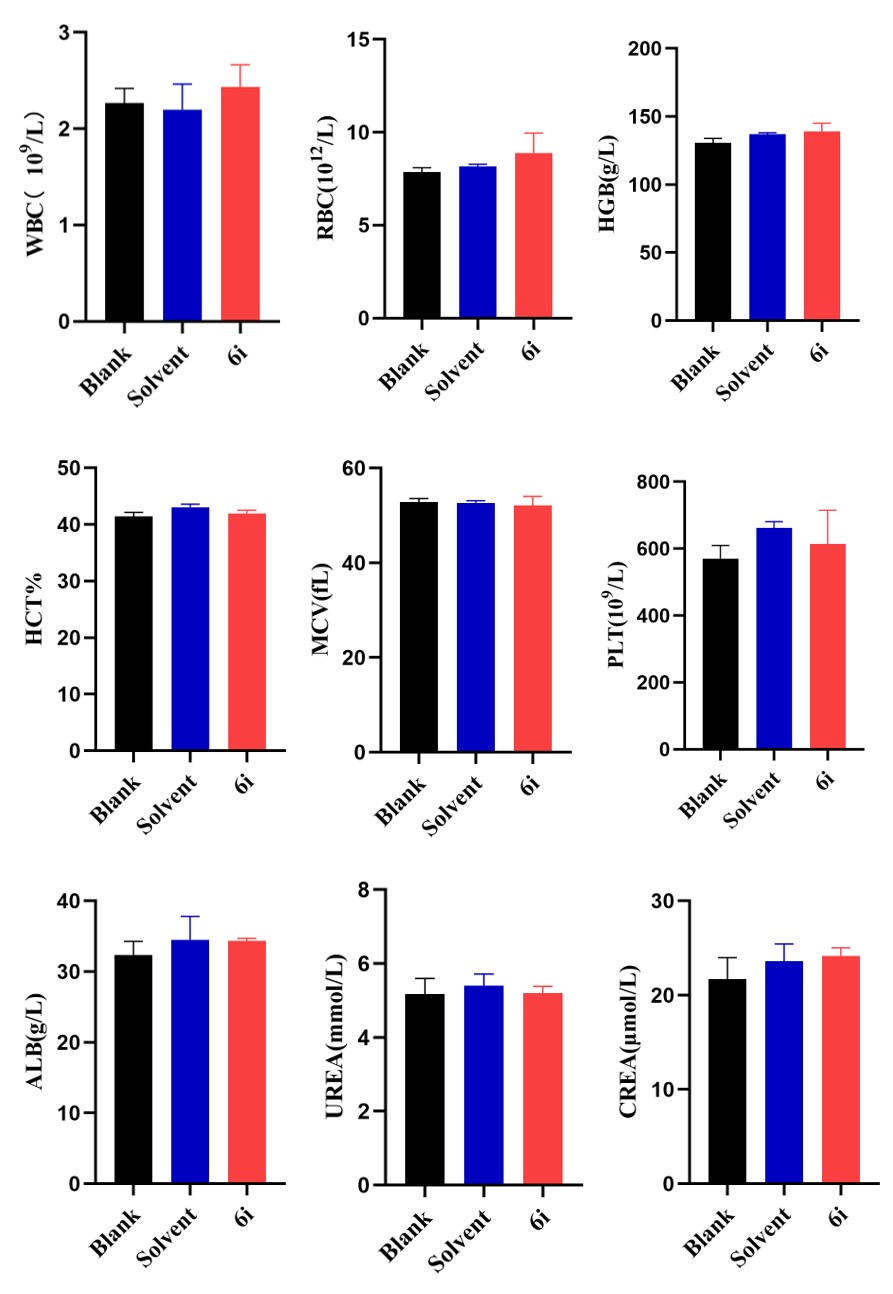


**Figure S5.** Routine blood testing and blood biochemical indexes in mice treated with compound 6i. WBC, white blood cell; RBC, red blood cell; HGB, hemoglobin; HCT, hematocrit; MCV, mean corpuscular volume; PLT, platelet count; ALB, albumin; UREA, urea; CREA, creatinine. Data are expressed as the mean ± SD. Error bars are representatives of three independent experiments. P-values were calculated using ordinary one-way ANOVA. No significance was observed in these tests.


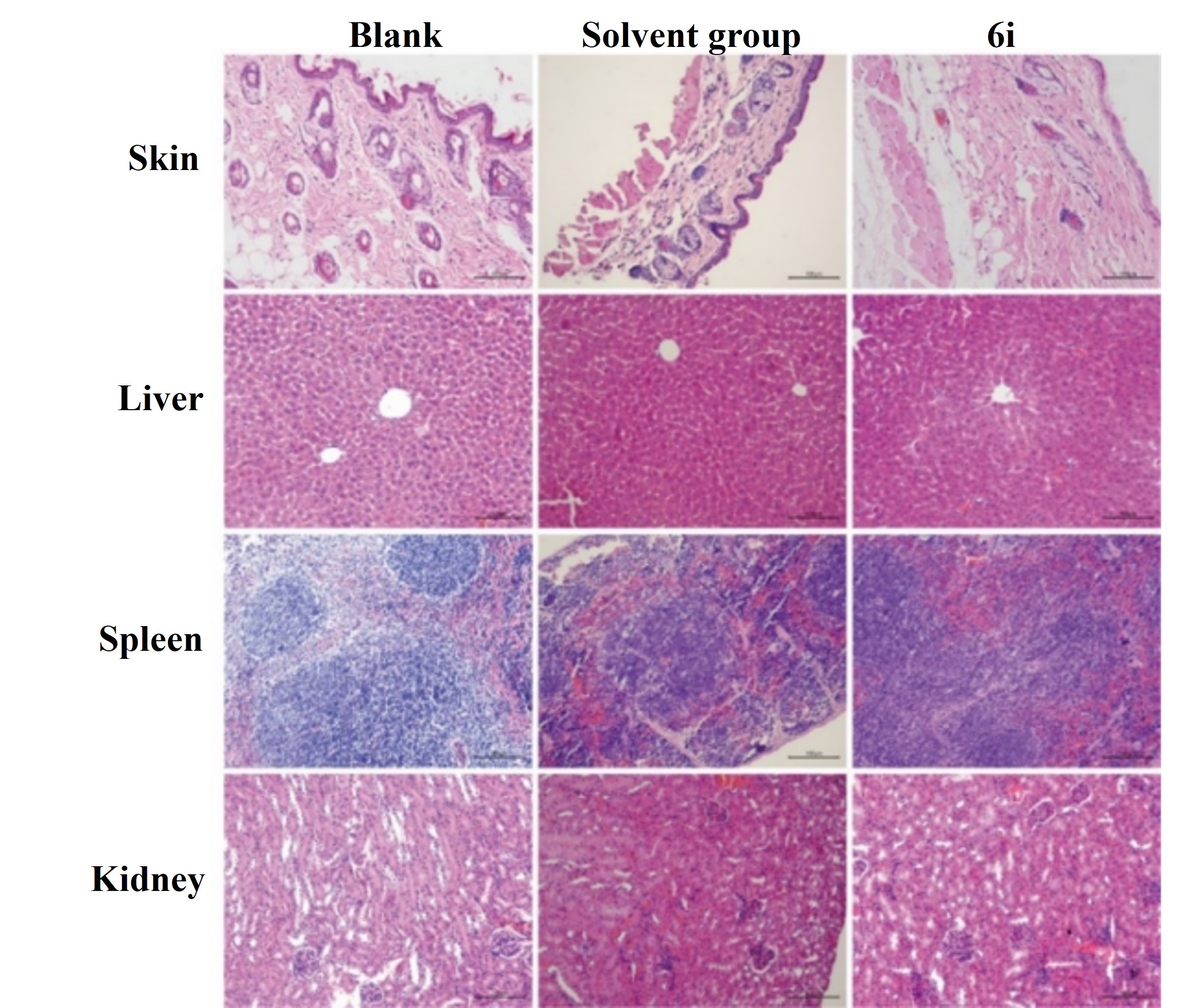


**Figure S6.** *In vivo* toxicity assay in normal KM mice treated with saline, 6i (10 mg/kg). Histological examination of the skin and major organs (liver, spleen, and kidney) resected from the mice (n = 5). Representative images were shown. Scale bar: 100 µm.


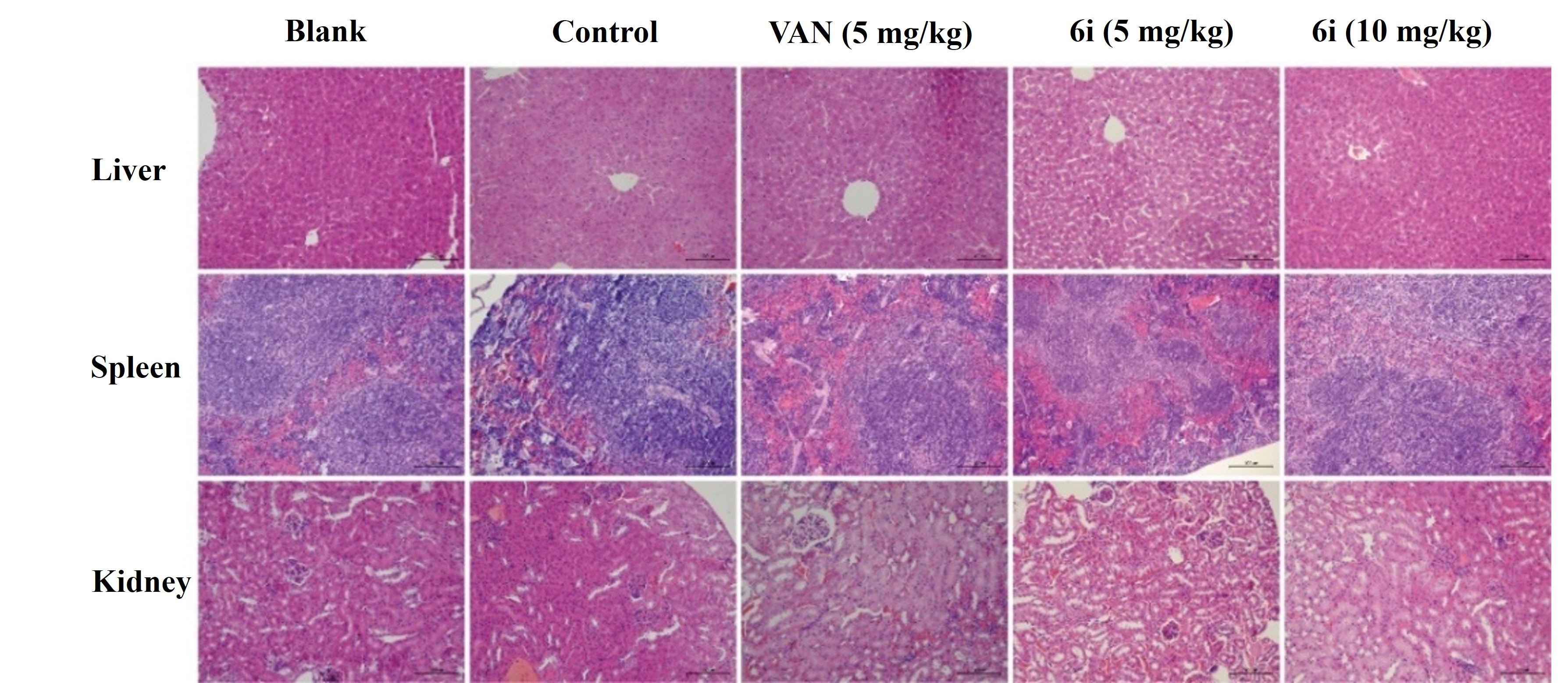


**Figure S7.** Representative H&E-stained sections from major organs after various treatments. Scale bar is 100 μm.
